# Supplementary material for: LINE-1 retrotransposons mediate cis-acting transcriptional control in human pluripotent stem cells and regulate early brain development
Source: Cell Genom. 2025 Aug 22;5(10):100979. doi: 10.1016/j.xgen.2025.100979 (PMC12791003; doi:10.1016/j.xgen.2025.100979)
Supplement: Document S1. Figures S1–S7 and Tables S1 and S2 [file mmc1.pdf]

Cell Genomics, Volume *5*

Supplemental information

LINE-1 retrotransposons mediate *cis*-acting transcriptional control in human pluripotent stem cells and regulate early brain development

Anita Adami, Raquel Garza, Patricia Gerdes, Pia A. Johansson, Fereshteh Dorazehi, Symela Koutounidou, Laura Castilla-Vallmanya, Diahann A.M. Atacho, Yogita Sharma, Jenny G. Johansson, Oliver Tam, Agnete Kirkeby, Roger A. Barker, Molly Gale Hammell, Christopher H. Douse, and Johan Jakobsson

Supplementary Figures

Figure S1.

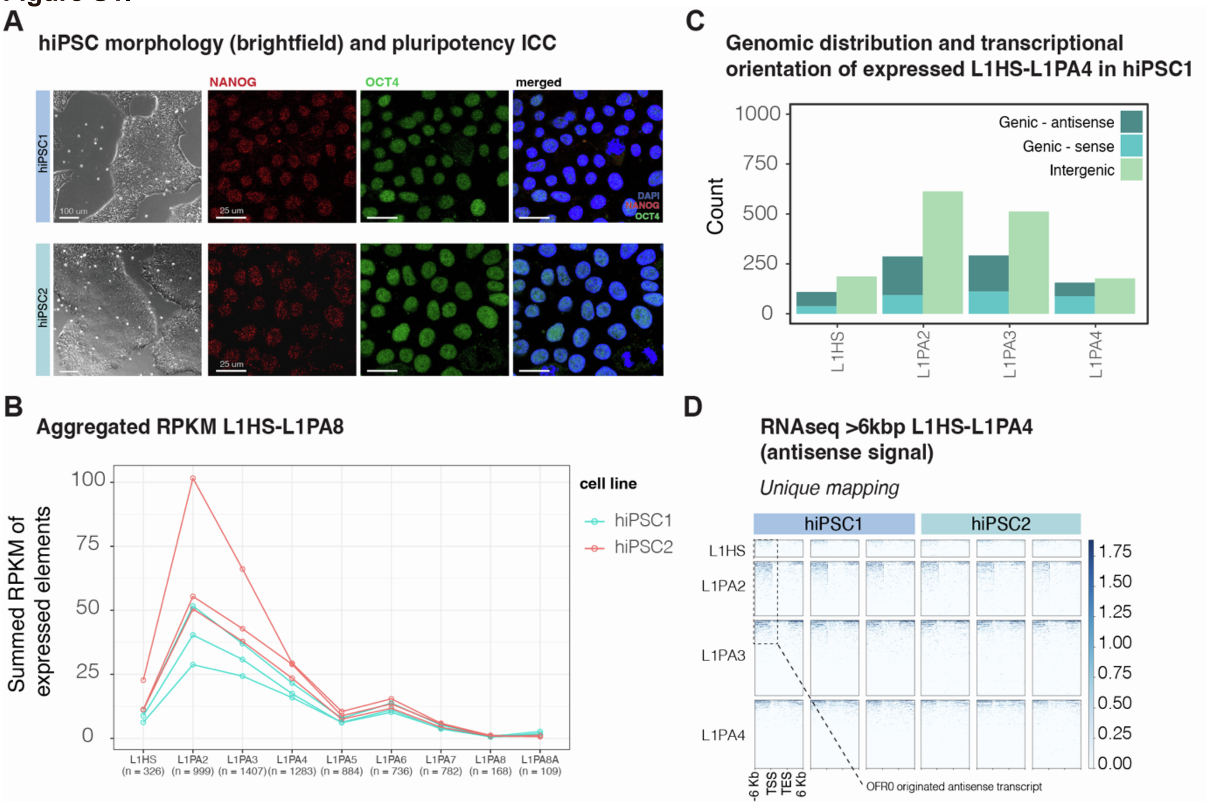

Supplementary Figure 1 **(related to Figure 1). L1s are highly expressed in hiPSCs.** A) Immunocytochemistry of the pluripotency marker NANOG (red), OCT4 (green) in hiPSCs. DAPI nuclear staining (blue) is included in the overlay. B) Line plot displaying expression levels of L1HS-L1PA8 subfamilies in hiPSC1 and hiPSC2. Data are presented as aggregated RPKM of the expressed elements at a subfamily level. C) Genomic distribution (genic/intergenic) and orientation relative to the host gene (genic: antisense/sense) of expressed L1HS-L1PA4 in the hiPSC1 cell line. D) Antisense expression of FL-L1s in two hiPSC lines. n= 3 technical replicates (normalized by RPKM).

Figure S2.

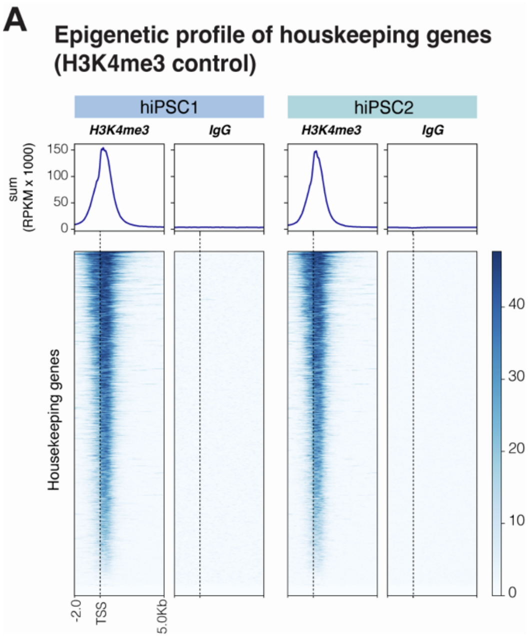

Supplementary Figure 2 **(related to Figure 2)*. *H3K4me3 CUT&RUN control.** A) CUT&RUN profiles of the active histone mark H3K4me3 over housekeeping genes (RPKM normalized). TSS = transcription start site. Profile plot at the top showing the summed signal.

Figure S3.

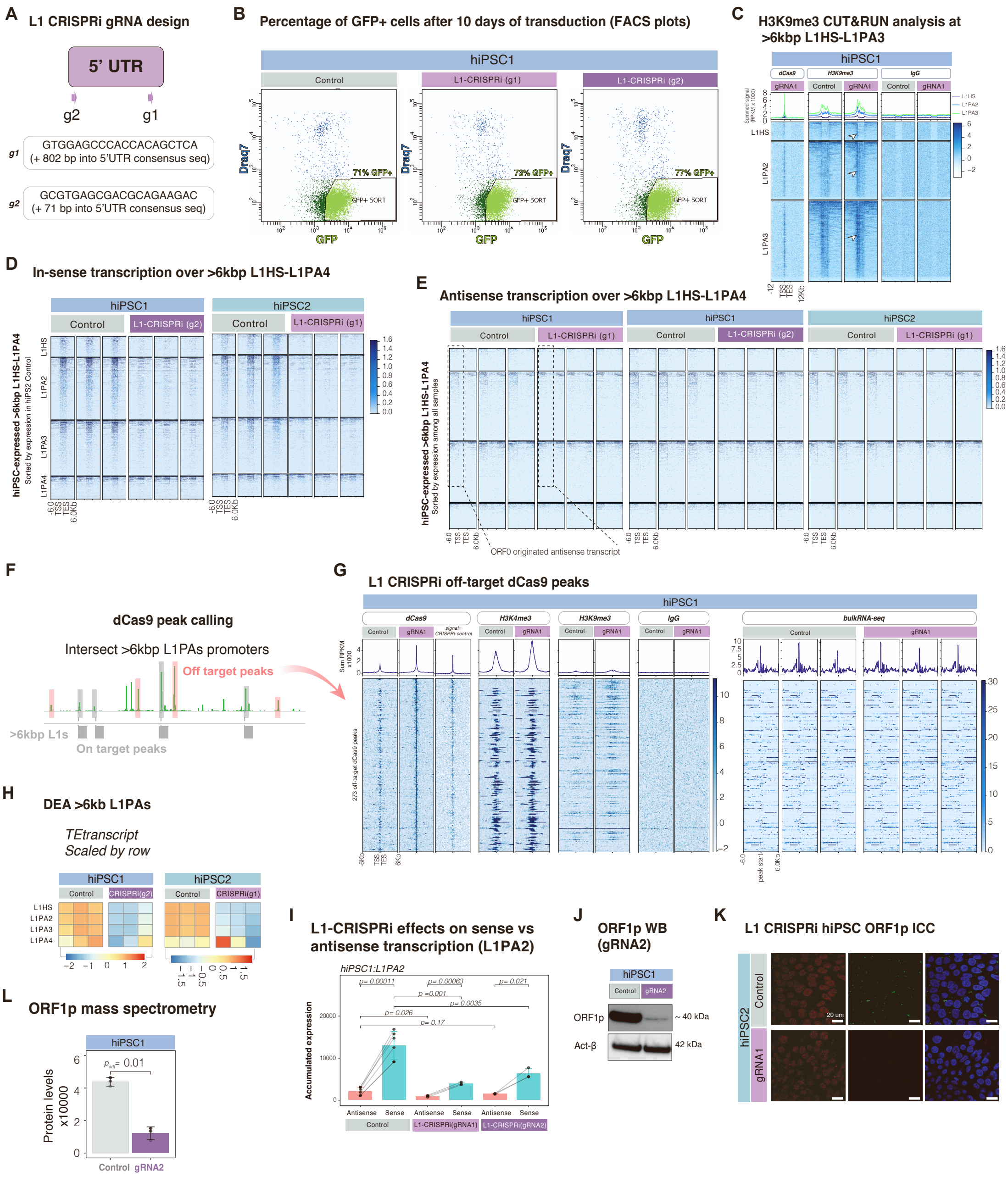

Supplementary Figure 3 **(related to Figure 3)*. *Efficient, on-target silencing of young, full-length L1s using CRISPRi.** A) Schematic of the gRNA target sites within the FL-L1. gRNAs designed to target the 5' UTR of L1 consensus sequence. B) FACS plots showing the percentage of GFP+ cells across the different conditions after 10 days of transduction with the CRISPRi constructs. C) CUT&RUN analysis of dCas9 binding and H3K9me3 over the targeted young, FL-L1s in control vs. L1 CRISPRi hiPSCs (RPKM normalized). Profile plot on top showing summed signal for L1HS-L1PA3 elements. IgG controls are reported on the right. D) Bulk RNA seq data showing the normalized expression of uniquely mapped, FL-L1s in L1-CRISPRi vs. control hiPSCs (all tracks RPKM normalized). E) Antisense expression (RPKM) of FL-L1s in control vs. L1 CRISPRi hiPSCs. Two hiPSC cell lines, two guide RNAs, three technical replicates each. F) Bioinformatical rationale to identify off-target effects based on dCas9 CUT&RUN. G) CUT&RUN showing dCas9 (gRNA1 - Control), H3K4me3, H3K9me3, IgG, and RNA-seq in control vs. L1 CRISPRi hiPSCs over off-target peaks (dCas9 peaks not overlapping with FL-L1s

promoters) (all tracks RPKM normalized). H) Expression of L1 families analysed using TEtranscripts in L1-CRISPRi vs. control hiPSCs in second gRNA and cell line (heatmap showing normalized expression, scaled by row). I) Barplot showing the L1-CRISPRi effects of gRNA1 and gRNA2 on sense or antisense transcription of the targeted L1PA2 elements. Bar representing mean normalized expression, error bars showing mean +/- standard deviation. J) Western blot (WB) of ORF1p (top) and ACTIN-β (bottom) in L1-CRISPRi using gRNA2 vs. control hiPSCs. K) Immunostaining on hiPSC2 of pluripotency marker NANOG (red) and L1-derived protein ORF1p (green) in L1-CRISPRi vs. control hiPSCs. DAPI nuclear staining in blue. L) Barplot displaying mass spectrometry data from hiPSC1 L1-CRISPRi (gRNA2) with ORF1p quantification. Bars indicate protein expression levels (x 1000) and error bars correspond to +/- standard deviation.

Figure S4.

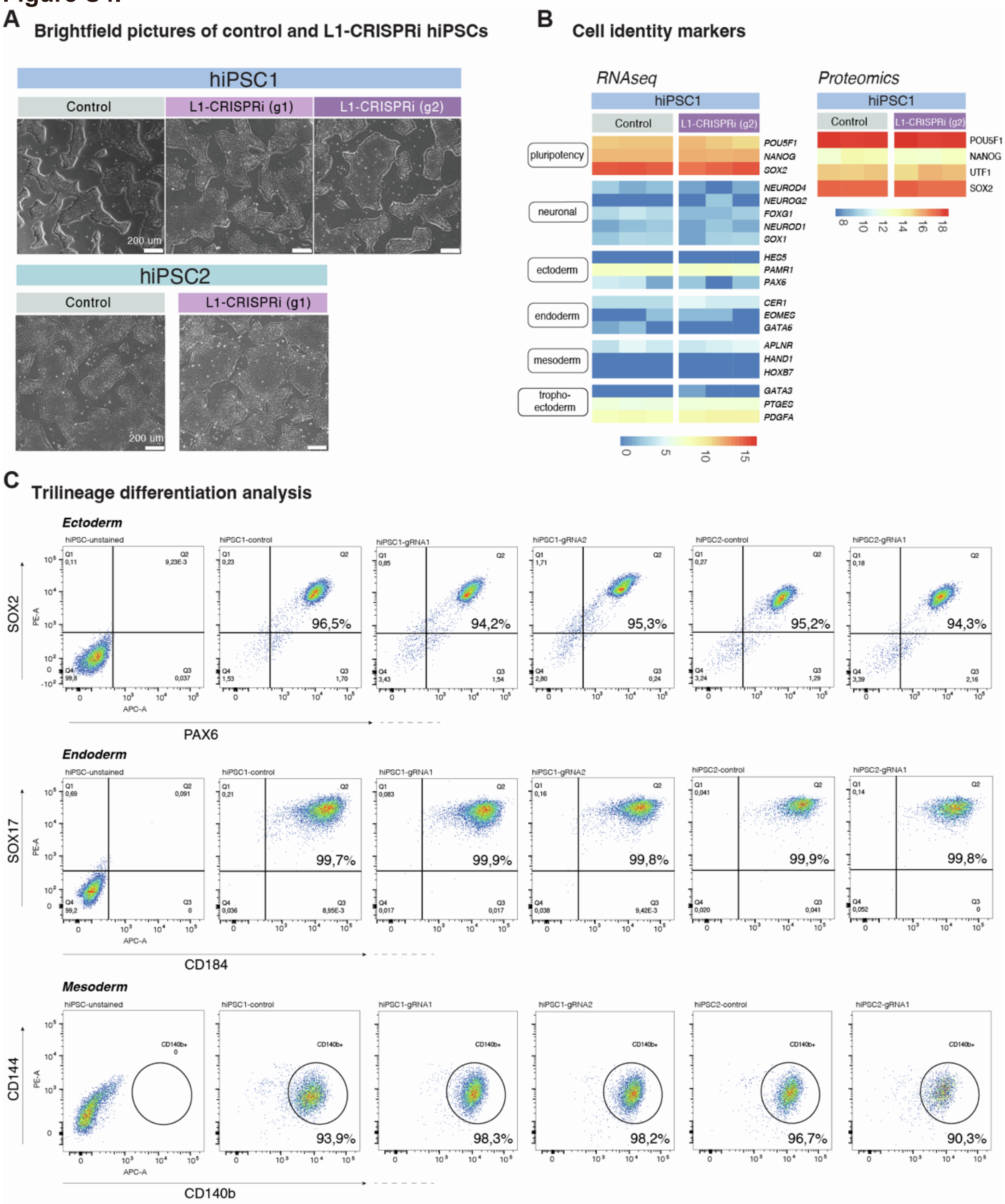

Supplementary Figure 4 **(related to Figure 3). L1-CRISPRi does not affect pluripotency.** A) Brightfield images of L1-CRISPRi hiPSC1 and hiPSC2 after transduction. Scale bar (white bar) is 200 μm. B) Cell identity markers, including pluripotency and cell differentiation markers, on a transcriptional (bulkRNA-seq) and on a protein level (mass spectrometry data). C) FACS plots displaying the results of the three germ-layer differentiation for all the transduced L1-CRISPRi hiPSC lines. Antibodies against SOX2 and PAX6 were used to define the ectoderm population; antibodies targeting SOX17 and CD184

were used to analyse the endodermal layer; anti-CD144 and anti-CD140b antibodies were used to define the mesoderm population. Percentage of positive cells is reported in each FACS plot.

Figure S5.

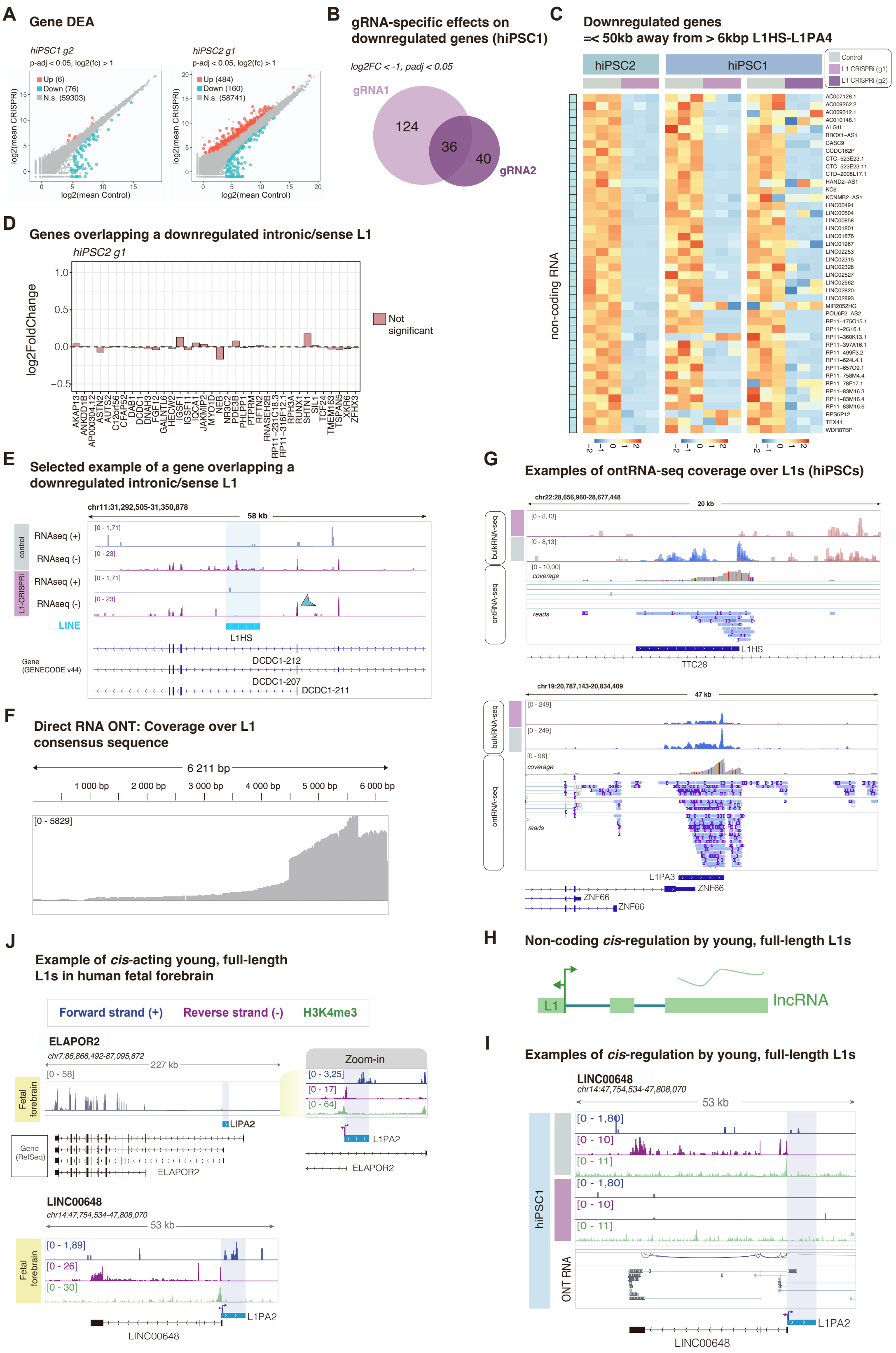

Supplementary Figure 5 **(related to Figure 4). L1s influence the expression of protein coding genes and long non-coding RNAs in *cis*.** A) Scatterplot showing mean gene expression in L1-CRISPRi (y-axis) and control (x-axis) hiPSCs using second gRNA and cell line. Plot showing

summarized results of differential expression analysis (DEA) (Bulk RNA-seq, n = 3 replicates / condition). Blue dots = significantly downregulated genes; red dots = significantly upregulated genes; grey dots = non-significant (DESeq2: Wald test; p-adj <0.05; log2(foldChange) >1). B) Venn diagram displaying gRNA-specific effects of gRNA1 and gRNA2 on the downregulated genes in L1-CRISPRi hiPSCs. C) Heatmap showing all the normalized expression of downregulated non-coding genes that overlap with a FL-L1 (n= 3 replicates / condition, heatmap showing log2 normalized expression). D) Log2FoldChange of expression of genes harboring an in-sense, downregulated (targeted) L1 upon L1-CRISPRi (hiPSC2 gRNA1). E) Genome Browser tracks showing the (unchanged) expression of the *DCDC1* locus and the downregulated, in-sense L1HS element silenced upon L1-CRISPRi. F) Direct RNA ONT reads mapped to L1 consensus sequence. G) Examples of ONT long-read RNA coverage over expressed L1s in hiPSCs. H) Schematic showing the expression of a L1-driven long non-coding RNA. I) Genome browser tracks showing the expression of an L1-driven long non-coding RNA in hiPSCs, and its silencing upon L1-CRISPRi. From top to bottom: control hiPSC bulk RNA-seq data (dark blue: forward transcription, purple: reverse transcription), H3K4me3 CUT&RUN (green, control vs L1-CRISPRi hiPSCs), L1-CRISPRi hiPSC bulk RNA-seq data, H3K4me3 CUT&RUN, and control hiPSC ONT direct RNA reads from control hiPSCs. J) Top: genome browser tracks showing the expression of *ELAPOR2* in human fetal forebrain. From top to bottom: bulk RNA-seq data in grey (left), zoom-in (right) showing bulk RNA-seq data (dark blue: forward transcription, purple: reverse transcription), and H3K4me3 CUT&RUN (green). Bottom: genome browser tracks showing the expression of *LINC00648* in human fetal forebrain. From top to bottom: bulk RNA-seq data (dark blue: forward transcription, purple: reverse transcription), and H3K4me3 CUT&RUN (green) (all tracks normalized by RPKM). Data from Garza *et al.* (2023)[26].

Figure S6.

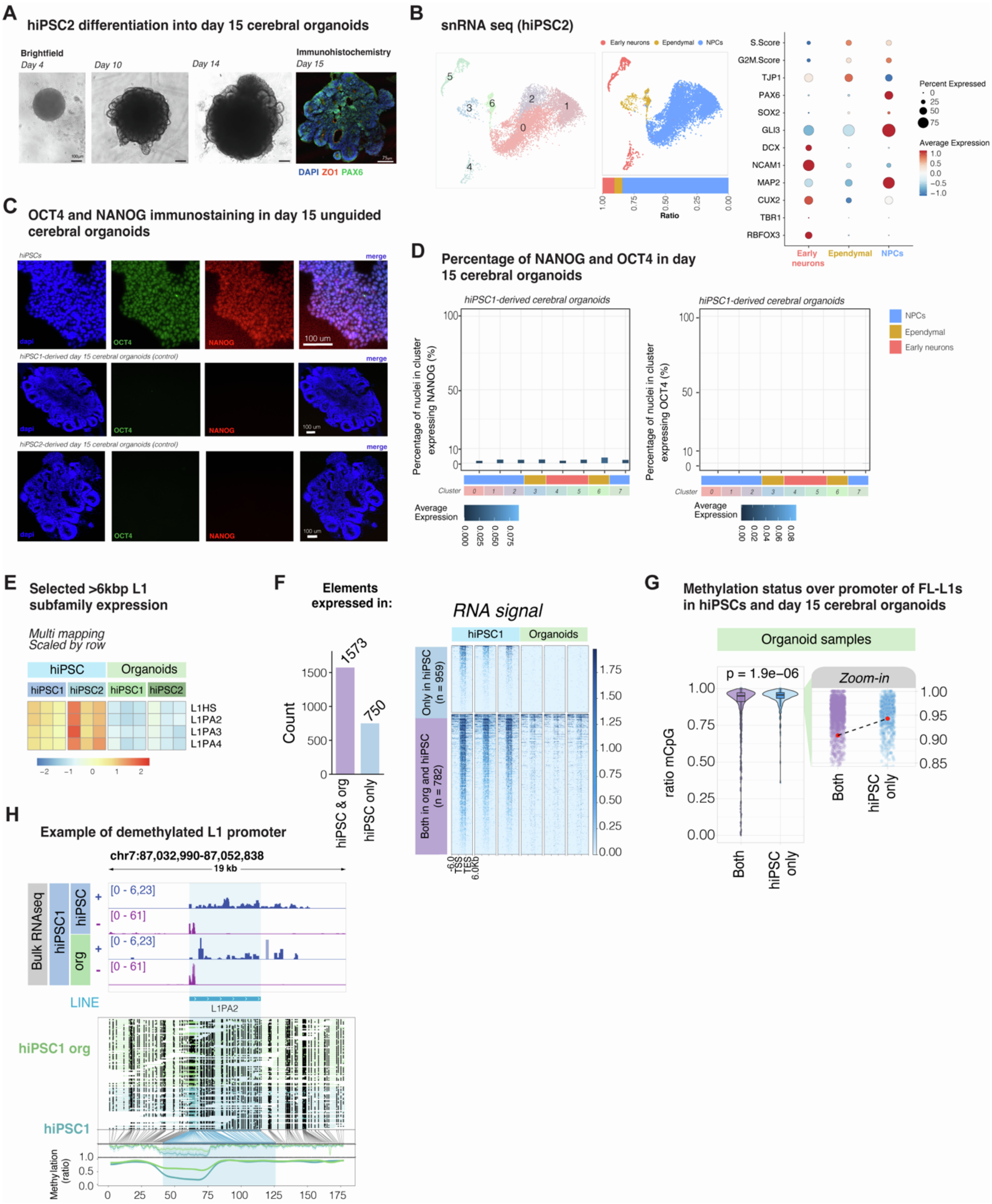

Supplementary Figure 6 **(related to Figure 5)*****.*** **Expression of evolutionary young full-length L1s in cerebral organoids.** A) Brightfield images of differentiating cerebral organoids (hiPSC2) at different time points and immunohistochemistry on day 15 (hiPSC2) organoids for ZO1 (red) and PAX6 (green). DAPI in blue. B) Left: UMAP showing clusters found in day 15 hiPSC2 unguided cerebral organoids. Middle: UMAP showing cell types found in day 15 hiPSC2 unguided cerebral organoids. Bar plot showing the percentage of the cell type composition of the cerebral organoids. Right: dot plot displaying selected neuronal and NPC markers used to characterize the cell clusters (dot size showing the percentage of cells expressing the gene, color indicates average expression in each cell type). C) OCT4 and NANOG immunostaining in day 15 unguided cerebral organoids. A positive hiPSCs control is included on top. Scale bar (white bar) is 100 μm. D) Percentage of nuclei from cerebral organoids expressing NANOG (left) or OCT4 (right) across different clusters at day 15 of differentiation in hiPSC1-derived cerebral

organoids. E) Expression of L1 families analyzed using TEtranscripts in L1-CRISPRi vs. control organoids and hiPSCs (heatmap showing normalized expression, scaled by row). F) Left: Barplot showing the number of expressed FL-L1 in hiPSC and day 15 cerebral organoids (purple), and hiPSC only (blue). Right: Bulk RNA seq data showing the normalized expression (RPKM) of FL-L1 in hiPSC and day 15 cerebral organoids (purple, bottom), and hiPSC only (blue, top) in hiPSC1 and day 15 hiPSC1-derived cerebral organoids (n = 3 replicates). G) Violin plots of the methylation status over the promoter of FL-L1 in hiPSC and cerebral organoids (purple), and hiPSC only (blue). Zoom-in panels indicating mean methylation levels (red dot). Boxplot centers correspond to the median, hinges correspond to the first and third quartile and whiskers stretch from the first and third quartile to + 1.5 IQR. H) Genome browser tracks showing normalized expression (RPKM) of L1s in hiPSCs and day 15 cerebral organoids (dark blue: forward transcription; purple: reverse transcription) and ONT DNA reads across day 15 organoids and hiPSCs. Black dots indicating methylated CpGs, and methylation coverage of the L1 elements at the bottom (green: organoids; blue: hiPSCs).

Figure S7.

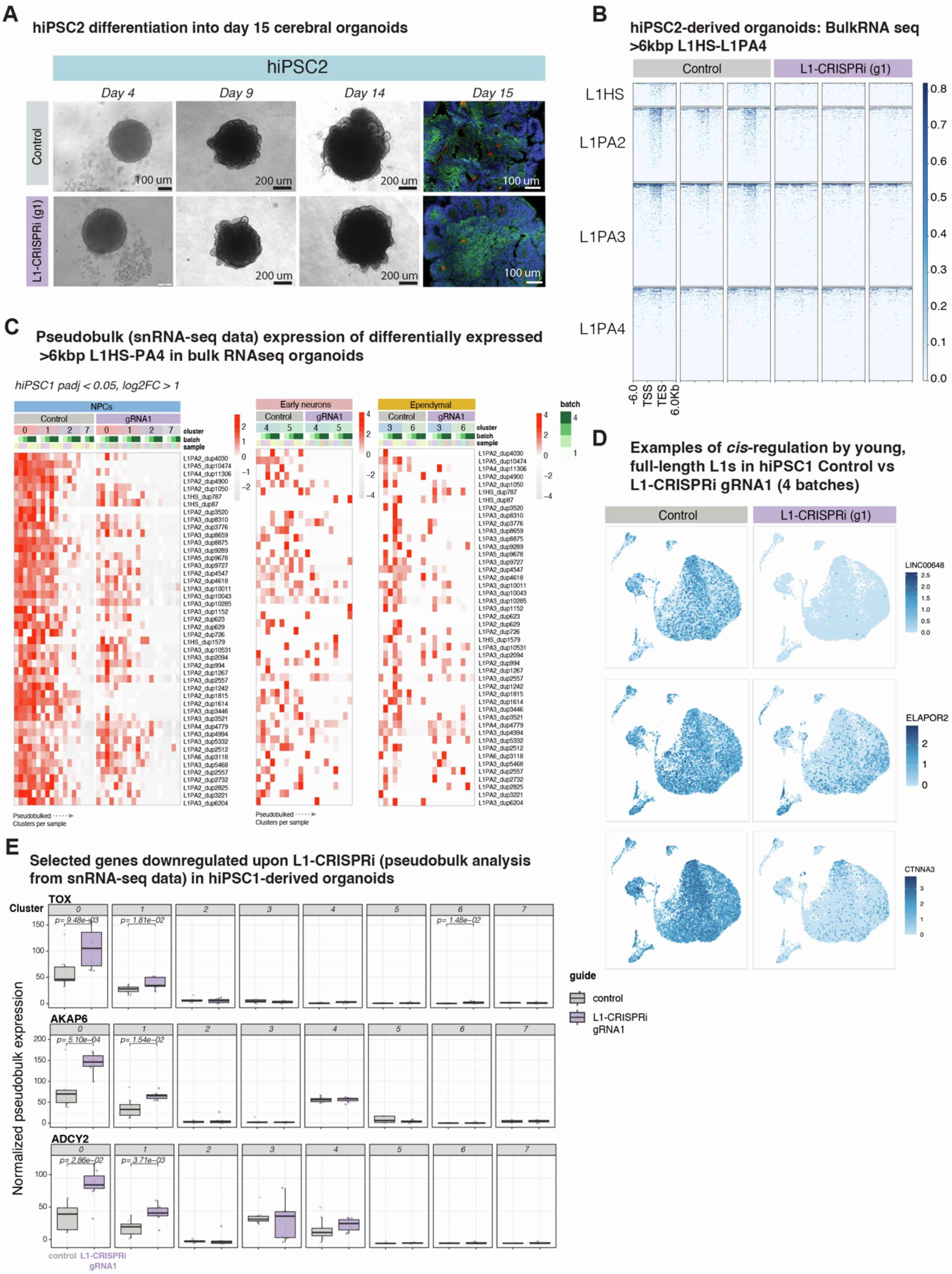

Supplementary Figure 7 **(related to Figure 6)*.*** A) Brightfield images of differentiating cerebral organoids (L1-CRISPRi and control hiPSC2) at different time points and immunohistochemistry on day 15 L1-CRISPRi and control hiPSC2 organoids for ZO1 (red) and PAX6 (green). DAPI in blue in the overlay. B) Normalized expression (RPKM) of uniquely mapped evolutionary young (L1HS-L1PA4), >6kbp L1s in control vs. L1-CRISPRi unguided cerebral organoids at day 15 of differentiation using hiPSC2 from bulk RNAseq data. C) Heatmap showing differentially expressed FL-L1s from bulk RNA-seq of L1-CRISPRi (control vs. gRNA1 hiPSC1) (DESeq2: Wald test; padj < 0.05, log2FC < -1) in snRNA-seq pseudobulks (n = 8 replicates (4 = gRNA1, 4 = control), in four organoid batches).

Expression is normalized by gene sizeFactors as calculated by DESeq2 (i.e. median of ratios) using pseudobulked gene expression. D) UMAPs showing L1-derived gene expression of selected candidates in control vs. L1-CRISPRi organoids (hiPSC1-derived organoids). E) Validation of changes in gene expression in L1-CRISPRi organoids using pseudobulk analysis. Boxplots showing the expression of selected downregulated genes upon L1-CRISPRi in day 15 cerebral organoids. Normalized pseudobulk expression shown on the y axis (Normalized by size factors using median of ratios, DESeq2). Boxplot centers correspond to the median, hinges correspond to the first and third quartile and whiskers stretch from the first and third quartile to + 1.5 IQR.

Supplementary Tables.

Table S1.

***Supplementary Table 1* (related to Figure 6G).** Gene names in order of appearance as shown in heatmaps of Figure 6G.

Downregulated	Upregulated
**LINC01891**	WDR31
**CSMD2**	GRIK1
**ZNF385B**	NR2F2−AS1
**LGI1**	AC087477.2
**CNTNAP2**	NTRK2
**MTUS2**	MLLT3
**LRRC7**	FILIP1
**LINGO2**	NDST4
**CACNA2D3**	FAT4
**AP002989.1**	ZNF536
**RNF217**	ADAMTSL1
**CTNNA3**	EPHA3
**LRRTM3**	TOX
**PIK3R1**	NR2F1
**AC007923.4**	AC027601.6
**SLC7A8**	PTCH1
**PAX7**	FEZF1
**CDON**	LINC00664
**LMX1A**	TLL2
**AC087854.1**	AL157944.1
**DOCK8**	SCN3A
**FSTL4**	EPHA5
**MYO5B**	PID1
**NOS2**	CNR1
**AP002387.2**	AKAP6
**PCAT1**	ADCY2
**PRNCR1**	CRABP1
**LINC01801**	CCKBR
**AC008555.4**	TMSB4X
**AC005537.1**	SLC38A2
**LAMA2**	UQCRFS1
**FAM183A**	LBH
**AC060809.1**	C19orf81
**AL513318.2**	SEMA3D
**AL442636.1**	SYTL4
**AC110760.1**	PLCB2
**DISC1FP1**	AC005740.5
**AC021504.1**	SCN1A

**AC079052.1**	GRB14
**AC106798.1**	FN1
**AC112206.2**	ADCY7
**AC011997.1**	
**TNFAIP8L3**	
**GLDN**	
**MIR4713HG**	
**CYP19A1**	
**LINC01376**	
**AC022031.2**	
**Z93403.1**	
**LINC01905**	
**AC009271.1**	
**LINC01416**	
**AC009262.1**	
**NPSR1−AS1**	
**XDH**	
**ARHGAP25**	
**ENPEP**	
**LRRTM4**	
**AC009315.1**	
**FSIP2**	
**SPINK5**	
**LINC02315**	
**LINC01876**	
**AC104041.1**	
**CASC9**	
**LINC02428**	
**KIAA1324L**	
**LINC01456**	
**IGSF1**	
**MASP1**	
**AC098656.1**	
**AL135784.1**	
**LINC02253**	
**LINC02254**	
**AC068051.1**	
**VWC2L**	
**PMFBP1**	
**TRIM5**	
**AC005009.2**	
**PDE1C**	
**NRP1**	

**PARM1**	
**EBF1**	
**XKR7**	
**AL033530.1**	
**LINC02542**	
**AC026341.1**	
**AC006206.2**	
**AC106799.2**	
**DLEU1**	
**LINC01980**	
**LINC01967**	
**AGMO**	
**SLCO1A2**	
**CPNE7**	
**CCDC192**	
**AL442125.1**	
**MYO1D**	
**PPP2R2C**	
**PLCG2**	
**RNF181**	
**LINC00958**	
**FAM189A2**	
**AC010896.1**	
**C14orf39**	
**AC092142.1**	
**SYNPR−AS1**	
**SYNPR**	
**TPRG1**	
**STS**	
**AC233296.1**	
**AP000829.1**	
**AL157778.1**	
**AC009312.1**	
**INMT**	
**CRHR2**	

Table S2.

Supplementary Table 2* (related to Figure 6)*. Number of nuclei per cluster in organoids.

Cell line	Cluster	Condition	Num of nuclei
**hiPSC2**	0	gRNA1	4200
**hiPSC1**	0	gRNA1	9643
**hiPSC2**	0	gRNA2	1712
**hiPSC1**	0	gRNA2	5481

**hiPSC2**	0	LacZ	4627
**hiPSC1**	0	LacZ	8794
**hiPSC2**	1	gRNA1	2414
**hiPSC1**	1	gRNA1	4249
**hiPSC2**	1	gRNA2	1029
**hiPSC1**	1	gRNA2	2092
**hiPSC2**	1	LacZ	2625
**hiPSC1**	1	LacZ	3935
**hiPSC2**	2	gRNA1	203
**hiPSC1**	2	gRNA1	1443
**hiPSC2**	2	gRNA2	126
**hiPSC1**	2	gRNA2	1278
**hiPSC2**	2	LacZ	1004
**hiPSC1**	2	LacZ	1917
**hiPSC2**	3	gRNA1	162
**hiPSC1**	3	gRNA1	1009
**hiPSC2**	3	gRNA2	80
**hiPSC1**	3	gRNA2	838
**hiPSC2**	3	LacZ	250
**hiPSC1**	3	LacZ	1033
**hiPSC2**	4	gRNA1	198
**hiPSC1**	4	gRNA1	822
**hiPSC2**	4	gRNA2	106
**hiPSC1**	4	gRNA2	284
**hiPSC2**	4	LacZ	146
**hiPSC1**	4	LacZ	648
**hiPSC2**	5	gRNA1	2
**hiPSC1**	5	gRNA1	404
**hiPSC2**	5	gRNA2	5
**hiPSC1**	5	gRNA2	323
**hiPSC2**	5	LacZ	781
**hiPSC1**	5	LacZ	490
**hiPSC2**	6	gRNA1	22
**hiPSC1**	6	gRNA1	174
**hiPSC2**	6	gRNA2	22
**hiPSC1**	6	gRNA2	535
**hiPSC2**	6	LacZ	373
**hiPSC1**	6	LacZ	103
**hiPSC2**	7	gRNA1	0
**hiPSC1**	7	gRNA1	311
**hiPSC2**	7	gRNA2	0
**hiPSC1**	7	gRNA2	14
**hiPSC2**	7	LacZ	0
**hiPSC1**	7	LacZ	299
